# Analyzing Workload-Monitoring Consistency and Same-Week Injury-Classification Performance in Female Adolescent Swimmers: An Exploratory Session-RPE and Neural Network Study

**DOI:** 10.3390/life16091461

**Published:** 2026-08-31

**Authors:** Xiaodong Zhao, Chunlei Li, Junqi Wu

**Affiliations:** 1 Chengdu University of Technology, Chengdu 610059, China; 2 School of Physical Education and Sport Training, Beijing Sport University, Beijing 100084, China

**Keywords:** session-RPE, training load, sports injury, adolescent swimmers, neural network, same-week injury classification

## Abstract

**Objective:** This exploratory study aimed to: (1) verify whether session-RPE (sRPE) can serve as a reliable workload-monitoring indicator in female adolescent swimmers aged 13–17 years; (2) compare the same-week injury-classification performance of sRPE-, exponentially weighted moving average (EWMA)-, and robust exponential decreasing index (REDI)-derived indicators using a multilayer perceptron (MLP) model; and (3) identify candidate indicator combinations for future prospective validation in youth swimming. **Design:** A 12-week prospective observational tracking study was conducted with 20 female adolescent 200 m individual medley swimmers (aged 13–17 years) from a single provincial youth swimming team (Zhejiang). Twenty-five injury events were recorded across 240 athlete-weeks. Because repeated athlete-weeks were analyzed as independent samples and injury status was classified within the same week as the load indicators, the classification findings are preliminary internal-validation results that may overestimate model performance; they are not externally validated prospective predictions. **Methods:** Pearson and Spearman correlation analyses, intraclass correlation coefficient (ICC), and Bland-Altman plots were used to evaluate associations and agreement between sRPE-derived indicators and traditional training-load indicators (swimming distance and training duration). Twelve sRPE-derived indicators (four each from sRPE, EWMA, and REDI) were used as input features for MLP same-week binary classification models. REDI was included because its natural-logarithmic weighting emphasizes long-term cumulative load, complementing the recency-weighted EWMA. Model performance was evaluated using area under the ROC curve (AUC), with Bootstrap resampling (1000 iterations) to characterize internal stability. **Results:** Swimming distance, which shares no computational component with sRPE, was very strongly correlated with sRPE (r = 0.965, *p* < 0.001). Training duration was also strongly correlated with sRPE (r = 0.972, *p* < 0.001), but this duration correlation is partly part-whole by construction because duration enters the sRPE calculation. After Z-score standardization, the ICC between duration and sRPE reached 0.614 (substantial agreement), although standardization does not imply direct interchangeability. Among the 12 single indicators, EWMA uncoupled ACWR had the highest observed AUC (0.946). The EWMA four-indicator combination (coupled ACWR + uncoupled ACWR + Diff + SD) had the highest observed internal AUC (0.958). Uncoupled ACWR and Diff indicators showed numerically higher AUCs than their coupled and SD counterparts, respectively; these differences were not formally tested statistically and confidence intervals overlapped. **Conclusions:** In this small, single-team, single-event sample of female adolescent 200 m individual medley swimmers, sRPE showed moderate-to-substantial agreement with traditional workload indicators. The EWMA four-indicator model showed the highest observed internal discriminative ability for same-week injury classification (AUC = 0.958), with EWMA uncoupled ACWR the highest-ranked single indicator (AUC = 0.946). These findings are preliminary because of the small number of injury events, class imbalance, athlete-level clustering, binary injury labels, absence of simpler-model comparison, and lack of lagged or external validation. The exploratory two-tier warning thresholds (yellow > 0.5, red > 0.7) are conceptual, non-optimized boundaries and require prospective derivation and validation in independent cohorts before any operational use.

## 1. Introduction

RPE (Rating of Perceived Exertion), first proposed by Borg, quantifies exercise intensity through athletes’ subjective perception [1] and was later incorporated into Banister’s Training Impulse framework for internal-load monitoring [2].

Foster’s session-RPE (sRPE) multiplies a modified CR-10 rating by session duration and is convenient, non-invasive, and low cost, making it suitable for settings with limited equipment resources [3,4].

As a subjective training load quantification tool, sRPE is widely used in various competitive sports. Its derived indicators, including the Acute:Chronic Workload Ratio (ACWR), training monotony, and training strain, can more accurately reveal the correlation between dynamic load changes and injury risk [5]. ACWR is the most commonly used injury risk assessment indicator in sRPE practice [6]. Gabbett proposed that when ACWR is in the range of 0.8–1.3, athletes are at relatively low injury risk (the ‘green zone’); when ACWR exceeds 1.5, injury risk increases significantly; and when ACWR exceeds 2.0, non-contact injury rates increase by 5–7 times [5,7].

However, coupled ACWR (in which the acute load forms part of the chronic load denominator) has well-documented mathematical limitations. Because acute load (1-week total) is a component of chronic load (4-week average), the coupled calculation introduces spurious correlation between the numerator and denominator, artificially compressing load variability and masking genuine load changes [8,8]. When training load decreases (e.g., during tapering or recovery periods), coupled ACWR paradoxically increases, creating false injury risk signals. Moreover, injury exhibits a lag effect where high load in one week may cause injury in the following week, yet coupled ACWR dilutes this signal by incorporating the current week’s load into the chronic load denominator [9,10]. It should be noted that the validity of ACWR itself remains an ongoing scientific debate: some studies in professional soccer failed to replicate the ACWR-injury association [11], and simulation work has questioned whether mathematical coupling necessarily biases practical estimates. Comparisons of coupled and uncoupled formulations should therefore be interpreted within this contested context.

To address these limitations, researchers introduced the exponentially weighted moving average (EWMA), which assigns higher weights to recent training loads through an exponential decay function, making the time-series model more responsive to acute load changes [9]. EWMA has been reported to provide higher discriminative performance for injury likelihood than traditional rolling-average ACWR [12]. Additionally, the robust exponential decreasing index (REDI), which employs natural-logarithmic weighting and incorporates individual recovery capacity, has been proposed as a composite indicator for identifying overtraining states [13,14]. However, the relative effectiveness of EWMA and REDI compared with traditional ACWR remains an active area of investigation, with inconsistent findings across sports and populations [13,15].

Despite the extensive application of sRPE across sports, research remains concentrated on adult athletes and ball sports, with little evidence in adolescent swimmers aged 13–17 years. Adolescence involves rapid physiological development, and athletes of the same chronological age can differ substantially in biological maturation; growth spurts may transiently alter anthropometrics, coordination, workload tolerance, and injury susceptibility, while also increasing variability in subjective RPE reporting [16]. Because the present study did not measure peak height velocity, menarcheal status, Tanner stage, or another maturation marker, chronological age alone cannot fully account for this developmental heterogeneity [17]. This gap supports the need for youth-specific, low-cost workload monitoring and for time-weighted metrics that can smooth transient fluctuations without obscuring genuine load surges.

Furthermore, existing injury-focused research in swimming has largely relied on traditional statistical methods such as logistic regression and ROC analysis. Machine-learning approaches capable of representing non-linear relationships among multiple load indicators remain insufficiently examined in adolescent swimmers. Importantly, the present analysis evaluates same-week classification of recorded injury occurrence; it does not establish whether workload indicators prospectively predict injuries in future weeks [18]. The relative classification value of coupled versus uncoupled ACWR and Diff versus SD indicators, and their candidate combinations, has not been systematically investigated in this population [19].

Based on these gaps, this exploratory study conducted a 12-week prospective observational tracking investigation with 20 female adolescent swimmers (aged 13–17 years, 200 m individual medley specialists) from the Zhejiang Provincial Youth Swimming Team. The study examined associations and agreement between sRPE-derived indicators and traditional workload indicators, then used an MLP model to compare same-week injury-classification performance across 12 individual indicators and 4 indicator combinations. The aim was to generate hypotheses and identify candidate low-cost monitoring metrics for future multi-center validation, rather than to provide a clinically validated prediction tool.

The study tested the following exploratory hypotheses: (1) sRPE-derived indicators would show acceptable consistency with traditional training-load indicators; (2) EWMA-derived indicators would show higher observed AUCs than traditional sRPE and REDI indicators for same-week injury classification; and (3) uncoupled ACWR and Diff indicators would show numerically higher AUCs than coupled ACWR and SD indicators, respectively. Given the sample size, repeated athlete-week structure, and absence of external validation, these hypotheses and the MLP results are interpreted as proof of concept rather than evidence of definitive indicator superiority.

## 2. Methods

### 2.1. Participants

The subjects were 20 elite female adolescent athletes from a single provincial youth swimming team (Zhejiang). The characteristics of the athletes were as follows: mean age 15.2 years (range: 13–17 years, SD = 1.3), all specializing in 200 m individual medley, with an average training experience of 4.5 years (range: 3–6 years, SD = 0.8), all at national level two or above (Table 1). This study was conducted from 1 February 2026 to 1 May 2026. Recruitment from one team, one event, and one sex was intentional for feasibility but substantially restricts external validity; the findings should not be generalized directly to sprint swimmers, long-distance swimmers, male athletes, other age groups, or other competitive levels. No biological maturation measures (e.g., peak height velocity, menarcheal status, or Tanner stage) were collected.

Inclusion criteria: (1) systematic swimming training for at least 3 years; (2) minimum of 5 training sessions per week; (3) no major injury requiring extended training cessation during the study period; (4) informed consent obtained from athletes and their guardians. Exclusion criteria: (1) severe injury requiring surgical treatment within 3 months prior to the study; (2) cognitive or psychological conditions affecting subjective assessment capacity. The study protocol was approved by the Ethics Committee of Beijing Sport University (No. 2026241H). All participants and their guardians provided written informed consent before study commencement. Because all athletes were recruited from a single provincial youth team and specialized in one event, the external validity of the findings is inherently limited; this constraint is revisited in the Limitations Section.

**Table 1:** Basic information of the participants.

Characteristic	Value (N = 20)
Age (years), mean (range)	15.2 (13–17)
Height (cm), mean (SD)	164.5 (5.8)
Weight (kg), mean (SD)	54.2 (6.3)
BMI (kg/m^2^), mean (SD)	20.0 (1.6)
Training experience (years), mean (SD)	4.5 (0.8)
Training sessions per week	5–6
Event	200 m individual medley

### 2.2. Data Collection

**Collection of RPE:** Training duration was defined as the time from the official start of coach-prescribed training after the standardized warm-up to the end of training. Within 30 min after each daily training session, athletes independently reported RPE using Foster’s modified CR-10 scale. Before formal data collection, athletes completed a one-week familiarization protocol comprising: (1) group explanation of the CR-10 anchors using examples from recent sessions; (2) supervised post-session practice ratings on each familiarization day; and (3) individual corrective feedback when a rating was inconsistent with the session content. Formal test-retest reliability statistics were not computed, and day-to-day rating stability was monitored only informally; therefore, inter-individual differences in RPE interpretation and reporting maturity may remain and could affect all downstream analyses. Athletes were instructed not to discuss ratings with each other, and trained research personnel collected all data within a fixed 15–30-min post-training window [20].

The RPE-related collection indicators in this study included training impulse (TRIMP), training monotony, training strain, acute load, chronic load, and Acute:Chronic Workload Ratio as defined below [13,21,22]:
*Training Load (Session Load) = RPE × Session Duration (minutes)* *Weekly Training Load = sum of daily training loads within a week* *Acute Load (AL) = sum of training loads within the most recent week* *Chronic Load (CL) = average weekly training load of the previous four weeks* *Coupled ACWR = Acute Load (1 week)/Chronic Load (4-week average)* *Uncoupled ACWR = Acute Load (1 week)/Chronic Load (3-week average)* *Weekly Ratio of Workload Change (Diff) = percentage change in adjacent weekly load* *SD of Weekly Load = standard deviation of daily loads within a week*

Collection of training duration: Training duration began when athletes commenced the coach-designed training content after the standardized warm-up and ended when the coach signaled training completion. Pre-training warm-up and post-training cool-down/stretching were excluded to isolate the prescribed training stimulus and keep the duration denominator consistent across athletes. This definition differs from protocols that include warm-up and cool-down, may reduce comparability with studies using total session time, and may underestimate total session exposure [23,24].

Collection of swimming distance: The total swimming distance for each training session was recorded by the coaching team, including all swimming components (warm-up sets, main sets, drill sets, and cool-down swimming) [25,26].

Collection of injury records: Injury definition, classification, and mechanism were determined in accordance with the International Olympic Committee consensus statement [27]. Injury was defined as tissue damage or functional impairment resulting from training or competition, requiring medical intervention or resulting in training restriction. This consensus definition combines two ascertainment routes (medical attention and time loss), so the injury label is heterogeneous in severity. The same injury persisting until full recovery was counted as a single injury; full recovery was defined as complete, physician-confirmed return to unrestricted full training for at least one consecutive week. Injuries recurring after full recovery were counted as separate injuries. Diagnosis was jointly performed by two licensed physicians with at least three years of clinical experience. Team medical staff coded weekly injury occurrence as a binary variable (0 = no injury, 1 = new injury beginning that week); ongoing symptoms of a previously recorded injury were not re-coded. Severity grade, time-loss duration, affected tissue, and acute-versus-overuse mechanism were not recorded, precluding severity- or mechanism-specific analyses. Minor complaints managed without formal medical consultation may not have been captured, so the observed injury rate should be interpreted as a lower-bound estimate.

Missing data: no imputation was performed. Sessions with missing RPE or volume records were excluded pairwise from correlation analyses, yielding 261 valid session records. For weekly indicators, days without recorded load contributed zero load within weekly sums, and weeks with more than two missing days were excluded, yielding 240 complete athlete-weeks [26]. For REDI, days with missing workload received zero weight (w(i) = 0). This choice avoided inventing unobserved exertion values and preserved the observed-load history, but it may attenuate long-term cumulative-load estimates if missing days reflect unrecorded training rather than true rest. The amount, pattern, and potential mechanisms of missingness were not formally analyzed, and missing-data sensitivity analyses were not performed.

### 2.3. Indicator Calculation

With the wide application of sRPE in workload monitoring, researchers have developed various derived indicators such as training monotony and training strain based on the calculation method of sRPE, providing a comprehensive perspective for scientifically evaluating training load and health status. The main derived indicators analyzed in this study include:

Training Monotony is the ratio of weekly training load to its standard deviation [28], reflecting the daily fluctuation degree of training load. Lower training monotony usually indicates a more balanced training arrangement, while higher monotony may suggest insufficient load variation, potentially increasing overtraining risk.

Training Strain is the product of weekly training load and training monotony. It can more accurately quantify the combined effect of training load magnitude and load variability on athletes’ fatigue, providing strong support for injury risk assessment.

EWMA assigns higher weights to recent training loads through an exponential decay function (lambda decay factor), making the time series model more sensitive to acute load changes [9]. It has been widely used in monitoring dynamic load changes in competitive sports and has demonstrated excellent performance in associating acute fatigue and injury risk [12]. Formally, EWMA(t) = lambda × Load(t) + (1 − lambda) × EWMA(t − 1), where Load(t) is the training load on day t and lambda = 2/(N + 1) is the decay factor determined by the averaging window N; following Murray et al. [12], the acute EWMA used N = 7 days and the chronic EWMA used N = 28 days.

REDI is a composite indicator combining workload decrease rate and individual recovery capacity through natural logarithmic weighting, specifically designed to identify overtraining states [14]. REDI’s long-term memory effect accumulates past training loads over extended periods, making it suitable for chronic fatigue monitoring. Formally, REDI(t) = Sum over i of w(i) × Load(t − i), where i denotes the number of days before day t and the weights w(i) are proportional to 1/ln(i + 2), so that older loads contribute progressively less while never dropping to zero [14]; days without a recorded load were assigned zero weight (w(i) = 0).

For this study, three base indicator types (sRPE, EWMA, REDI) were each used to derive four features: coupled ACWR, uncoupled ACWR, Diff, and SD, producing 12 individual indicators. Because the features have heterogeneous units, all features were Z-score normalized before correlation analyses and MLP modeling. For MLP modeling, data from all 20 athletes were aggregated over the 12-week period (N = 240 athlete-weeks), with each athlete-week treated as an independent sample despite repeated observations within athletes. The 12 indicators served as input features, and same-week injury occurrence (yes/no) served as the binary output label. This design therefore evaluates concurrent classification rather than prediction of future injuries. Athlete-specific clustering (training history, injury susceptibility, technique, and maturation) was not modeled; indicators computed within each athlete partially absorb between-athlete level differences but do not remove within-athlete correlation.

### 2.4. Statistical Analysis

The experimental data were calculated using WPS Office 2024, and secondary processed data were analyzed using SPSS 27.0, with graphs drawn using GraphPad Prism 9.5.1. Measured study data are expressed as mean +/− SD.

The Shapiro-Wilk test was used to assess normality of all continuous variables (alpha = 0.05). Pearson correlation analysis was employed to examine relationships between training volume indicators (distance, duration) and training intensity indicators (sRPE, EWMA, REDI). Interpretation criteria: |r| > 0.8 indicates very strong correlation, 0.6 < |r| ≤ 0.8 strong, 0.4 < |r| ≤ 0.6 moderate, |r| ≤ 0.4 weak. Spearman rank correlation analysis was used as a robustness check.

To evaluate consistency, data were Z-score standardized to eliminate dimensional differences. ICC (two-way random effects model, single measure) was calculated for each indicator pair, interpreted following Landis and Koch [29] and Koo and Li [30]. Standardization improves comparability of measurement scales but does not establish that sRPE-derived and traditional workload indicators are interchangeable. Bland-Altman analysis was performed to assess systematic bias and limits of agreement between sRPE-derived indicators and traditional training-volume indicators.

For same-week injury classification, an MLP neural network binary classification model was constructed using SPSS 27.0. The MLP was selected as an exploratory non-linear classifier because workload-injury relationships may involve thresholds, interactions, and time-dependent accumulation rather than only linear main effects. To limit model complexity in this small dataset, the architecture was restricted to one hidden layer, and most comparative models used a single input feature. Nevertheless, with only 25 injury events and up to 12 inputs, the MLP remains vulnerable to overfitting. Because repeated athlete-weeks were treated as independent even though they were nested within athletes, the effective sample size is lower than the nominal 240 and model uncertainty may be underestimated. Regularized logistic regression, generalized additive models, mixed-effects models, and other simpler benchmarks were not fitted in this proof-of-concept analysis; consequently, the present results cannot establish that the MLP adds predictive value over simpler approaches. Benchmarking against penalized logistic regression and using athlete-aware resampling are therefore essential next steps.

The model architecture comprised one hidden layer with the number of hidden units determined by SPSS automatic architecture selection, hyperbolic tangent (tanh) activation in the hidden layer, a softmax output layer, and cross-entropy error; optimization used the scaled conjugate gradient algorithm with default SPSS convergence criteria. Reliance on automatic architecture selection reduces reproducibility because the selected hidden-unit count is not transparently reported here. No oversampling, undersampling, or class weighting was applied. Input features were Z-score normalized, and the dataset was partitioned once using a 7:3 training-to-test ratio. AUC was used as a rank-based measure of discriminative ability and categorized descriptively as high (≥0.80), moderate (0.60–0.79), or weak (<0.60); these categories are not sensitivity at a fixed threshold. Because injury weeks comprised only 10.4% of observations, AUC alone does not assess calibration, precision, recall, F1 score, or clinical utility. Bootstrap resampling (1000 iterations) was used to estimate 95% confidence intervals, but a single 70/30 split remains split-dependent; repeated k-fold cross-validation, cluster-robust bootstrap validation, or leave-one-athlete-out validation would provide stronger robustness assessment.

Twelve single-indicator models (each with one derived indicator as input) and four combination models (sRPE four-indicator, EWMA four-indicator, REDI four-indicator, and all-12-indicator combined) were constructed for same-week injury classification and compared descriptively using AUC and Bootstrap confidence intervals.

## 3. Results

The 12-week tracking study yielded 261 valid training observation records across 20 athletes; aggregated by week, 240 athlete-weeks were available for same-week classification modeling, of which 25 weeks contained a newly recorded injury event (10.4%) and 215 were injury-free. Because these athlete-weeks are nested within only 20 athletes, the nominal sample size overstates the amount of independent information and AUC estimates may be optimistic. This severe class imbalance limits threshold-dependent interpretation and increases the risk of optimistic performance estimates. Descriptive statistics for the primary variables are presented in Table 2. Training distance showed considerable variability (CV approx. 47.5%), and training duration and sRPE had CV values of approximately 43.7% and 56.4%, respectively, reflecting differing load arrangements across training phases. EWMA’s CV (approx. 46.6%) was lower than that of raw sRPE, demonstrating smoothing. REDI’s larger mean value is consistent with the cumulative nature of its logarithmic weighting scheme.

The Shapiro-Wilk test indicated that all five primary variables deviated significantly from normality (*p* < 0.05). Because the underlying session-level values were not available for retrospective calculation, median and IQR could not be added to Table 2; the archived aggregate statistics are therefore retained despite the non-normal distributions. Pearson correlations were retained as the primary analysis for two reasons: with N = 261 the Pearson coefficient is robust to moderate deviations from normality, and Pearson coefficients permit direct comparability with the workload-monitoring literature, which reports Pearson almost exclusively. Spearman rank correlations were computed for every pair as a robustness check and agreed closely with the Pearson values (all differences below 0.03), confirming that the conclusions do not depend on the distributional assumption.

Pearson correlation analysis (Table 3) showed that swimming distance was very strongly positively correlated with sRPE (r = 0.965, *p* < 0.001), EWMA (r = 0.938, *p* < 0.001), and REDI (r = 0.734, *p* < 0.001). Because swimming distance has no computational component in common with sRPE, the distance-sRPE correlation is the primary empirical evidence supporting workload-monitoring validity. Training duration was also very strongly correlated with sRPE (r = 0.972, *p* < 0.001), EWMA (r = 0.956, *p* < 0.001), and REDI (r = 0.782, *p* < 0.001), but the sRPE-duration association is partly part-whole by construction because duration enters the sRPE calculation and therefore should not be interpreted as independent validation. All six pairings reached statistical significance (*p* < 0.01). Fisher z 95% confidence intervals are reported in Table 3.

**Figure 1:**
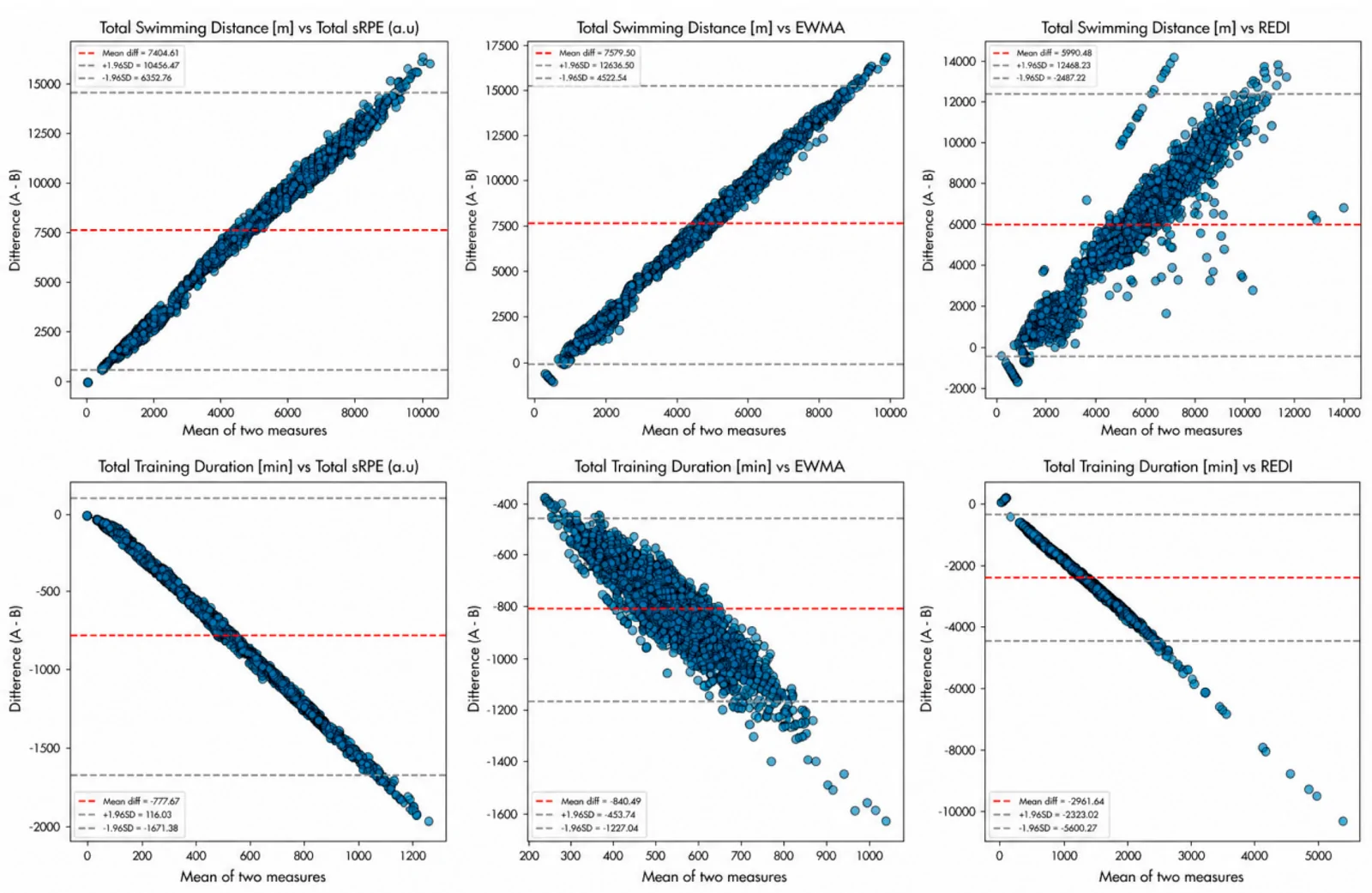
Bland-Altman analysis of all indicators.

ICC(0,1) analysis with Z-score standardized data showed that the duration-sRPE pairing achieved the highest agreement (ICC = 0.614, substantial agreement per Landis and Koch [29]; moderate-to-good per Koo and Li [30]), while the distance-REDI pairing had the lowest (ICC = 0.318, fair agreement). Overall, training duration demonstrated higher ICC values with sRPE-derived indicators than swimming distance, consistent with duration being a core component of sRPE. These standardized ICC values indicate scale-aligned trend agreement only; they do not imply that the indicators are directly interchangeable (Figure 1).

Bland-Altman analysis showed that most data points (>95%) fell within the 95% limits of agreement (Mean +/− 1.96 SD), with differences distributed on both sides of the mean-difference axis and no obvious funnel pattern. These findings suggest stable agreement at the trend level after standardization. However, no calibration equations or conversion models were developed; therefore, the results should not be interpreted as establishing predictable numerical conversion or interchangeability between sRPE-derived indicators and traditional workload measures (Figure 1).

### Neural Network MLP Same-Week Injury Classification Results

Twelve single sRPE-derived indicators were used as individual input variables for MLP binary same-week classification models and ranked by test-set AUC (Table 4). Six indicators reached the descriptive high-AUC category (AUC ≥ 0.80), three were classified as moderate (0.60–0.79), and three as weak (<0.60). These categories describe rank-based discrimination only and are not sensitivity at a fixed decision threshold.

EWMA uncoupled ACWR had the highest observed AUC (0.946), followed by EWMA Diff (0.906) and sRPE uncoupled ACWR (0.895). EWMA-based indicators occupied three of the four highest ranks. Uncoupled ACWR had numerically higher AUCs than coupled ACWR within the EWMA and sRPE families, and Diff indicators had numerically higher AUCs than SD indicators. However, several Bootstrap confidence intervals overlapped (for example, REDI coupled ACWR 0.741–0.945), no DeLong or other formal AUC comparisons were performed, repeated athlete-weeks were treated as independent, and only a single 70/30 split was used. The numerical ranking should therefore be interpreted as an exploratory directional pattern rather than confirmed superiority.

When the four derived features from each base indicator type were jointly input to the MLP model, the EWMA four-indicator combination had the highest observed AUC (0.958), followed by the all-12-indicator model (0.913), the sRPE four-indicator model (0.866), and the REDI four-indicator model (0.742). Because confidence intervals overlapped, no formal AUC comparisons were performed, athlete-weeks were clustered within athletes, and validation relied on a single internal split, these values should be interpreted as internal, exploratory estimates rather than proof of a statistically superior combination (Table 5).

**Table 5:** Indicator-combination MLP same-week injury classification results.

Model	AUC	SE	95% CI Lower	95% CI Upper	AUC Category
EWMA 4-indicator	0.958	0.026	0.907	1.000	High
All 12-indicator	0.913	0.038	0.839	0.987	High
sRPE 4-indicator	0.866	0.048	0.772	0.960	High
REDI 4-indicator	0.742	0.065	0.615	0.869	Moderate

**Figure 2:**
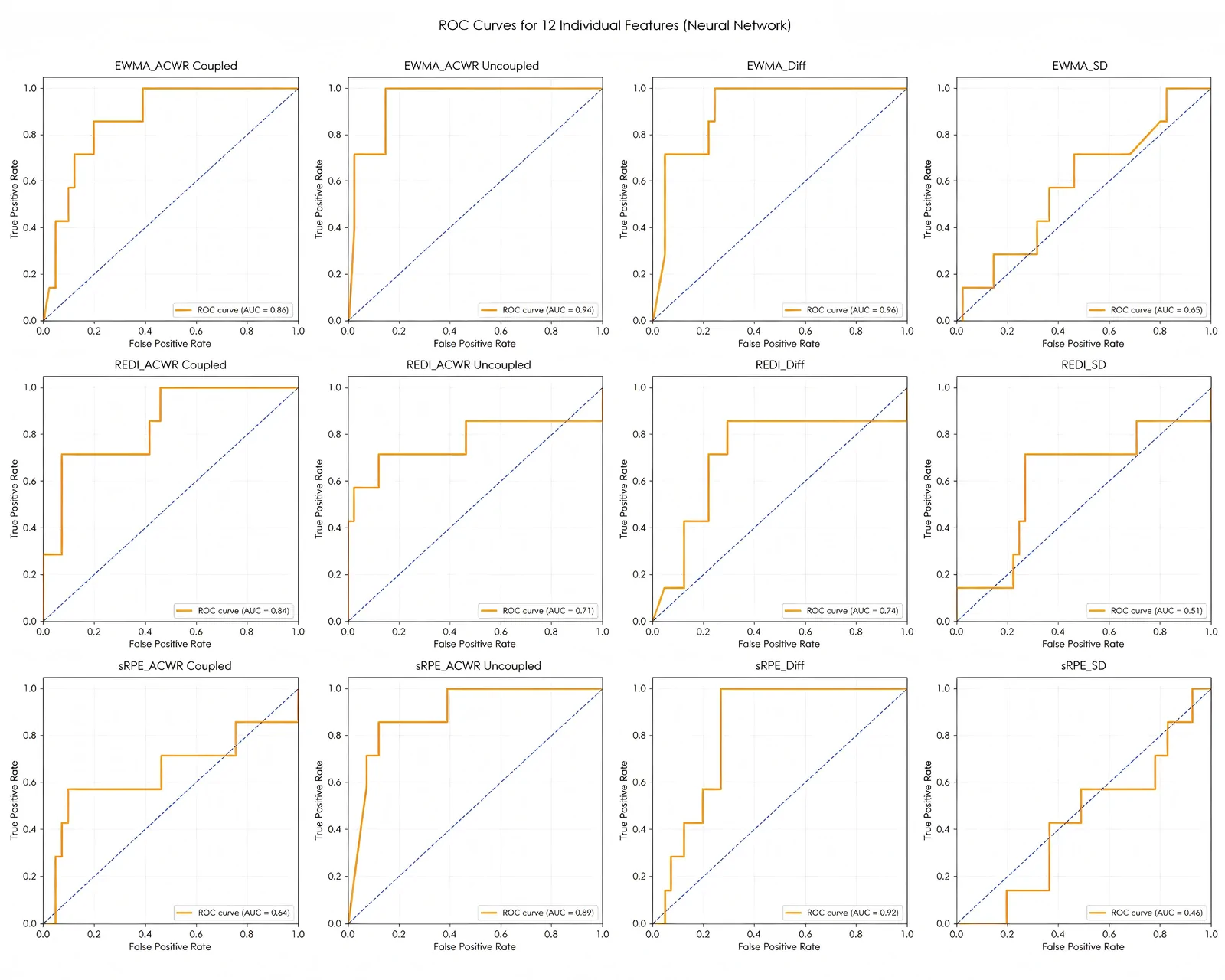
ROC curves of 12 indicators.

**Figure 3:**
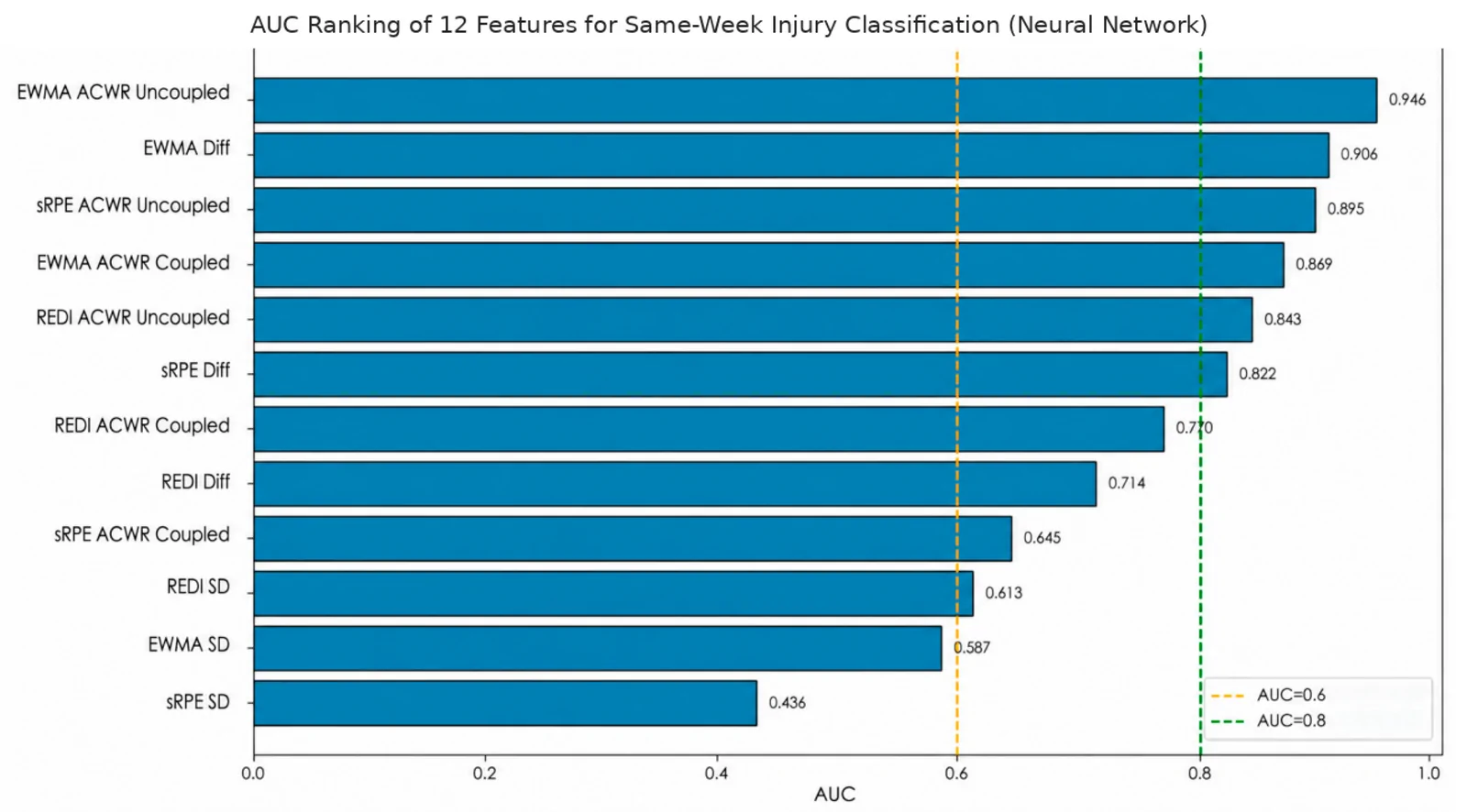
AUC ranking of 12 indicators.

The EWMA four-indicator combination conceptually covers four complementary dimensions: uncoupled ACWR (short-term load surge), coupled ACWR (longer-term load balance), Diff (week-to-week change velocity), and SD (within-week load dispersion). One possible explanation for the lower AUC of the all-12-indicator model is that low-discrimination or redundant features added noise and diluted information from the EWMA indicators. This interpretation is speculative because feature importance, feature redundancy, and model-selection statistics were not quantified (Figure 2 and Figure 3).

## 4. Discussion

The main findings are that sRPE was strongly associated with traditional workload indicators in this sample of female adolescent 200 m individual medley swimmers, and that EWMA-derived indicators showed the highest observed AUCs for same-week injury classification. Within the EWMA framework, uncoupled ACWR and Diff were the highest-ranked single indicators, and the EWMA four-indicator combination had the highest observed internal AUC (0.958). The second exploratory hypothesis was only partially supported: EWMA indicators occupied several top ranks, but REDI coupled ACWR (AUC = 0.843) exceeded some sRPE indicators, AUC differences were not formally compared, confidence intervals overlapped, repeated athlete-weeks were treated as independent, and no simpler benchmark model was fitted. Accordingly, all rankings represent exploratory directional patterns rather than confirmed superiority or prospective predictive performance.

### 4.1. Consistency of sRPE in Adolescent Swimmers

The results show that sRPE was very strongly correlated with traditional workload indicators. The distance-sRPE correlation (r = 0.965, *p* < 0.001) provides the stronger independent evidence because swimming distance has no computational component in common with sRPE. The duration-sRPE correlation was slightly higher (r = 0.972, *p* < 0.001), but session duration enters the sRPE calculation, making this association partly part-whole by construction rather than independent validation. Together with standardized ICC and Bland-Altman findings, these results support sRPE as a useful workload-monitoring tool within this specific population, while not establishing direct interchangeability with objective volume measures.

One possible explanation for these associations is that sRPE integrates physiological, metabolic, technical, and psychological fatigue signals [3,16]. The 200 m individual medley is metabolically mixed and technically demanding; however, energy-system contributions, cardiorespiratory strain, lactate responses, and neuromuscular fatigue were not directly measured. Therefore, interpretations involving glycolytic metabolism, fatigue accumulation, or adolescent physiological vulnerability should be regarded as hypotheses generated from the observed associations rather than mechanisms demonstrated by this dataset.

The slightly higher duration-sRPE than distance-sRPE correlation should also be interpreted cautiously because of the part-whole relationship. Swimming sessions include rest intervals, technical instruction, starts and turns, and recovery swimming that contribute to elapsed training time but not distance; these components may influence perceived exertion. Thus, duration may capture aspects of session demand not represented by distance, but the computational overlap with sRPE prevents the duration correlation from serving as independent evidence of validity.

ICC and Bland-Altman analyses indicate that standardized sRPE-derived indicators track the broad pattern of traditional workload measures but are not directly interchangeable. Post-standardization, the duration-sRPE ICC reached 0.614, and Bland-Altman plots showed that most observations fell within the 95% limits of agreement. Because no calibration equations were fitted, these findings do not establish numerical conversion between metrics. They instead suggest trend-level correspondence while leaving measurement-scale differences and potential bias unresolved.

### 4.2. Selection of Time Series Weights and Indicator Performance

EWMA-based indicators showed the highest observed AUCs among the three base indicator types in this dataset. A plausible explanation is that exponential weighting emphasizes recent loads while smoothing single-day noise, which may help capture short-term load changes relevant to injury risk in adolescents. However, this interpretation is speculative because physiological markers, wellness measures, and injury mechanisms were not collected, and the athlete-clustered sample was validated only internally.

Uncoupled ACWR had numerically higher AUCs than coupled ACWR across the base-indicator families. One possible explanation is that removing the acute week from the chronic denominator reduces mathematical coupling and preserves more load-variation signal. However, this interpretation remains speculative because formal statistical comparisons were not performed, the Bootstrap confidence intervals overlapped, and the models classified injuries within the same week rather than testing whether week N loads predict week N + 1 injuries.

Diff indicators had numerically higher AUCs than SD indicators, suggesting that week-to-week load change may carry more same-week classification information than within-week dispersion in this dataset. This pattern is consistent with the possibility that rapid load escalation is relevant to injury risk. However, injury mechanisms and lag times were not measured, so the finding should be treated as a hypothesis for future lagged analyses rather than evidence of a causal trigger.

REDI-based indicators showed lower observed AUCs overall (0.613–0.843). One possible explanation is that REDI’s long-memory logarithmic weighting may be less responsive to short-term fluctuations than EWMA in this dataset. However, this interpretation is speculative because acute-versus-overuse injury mechanism was not recorded. REDI may still be a useful candidate for hypotheses concerning chronic fatigue and cumulative-load monitoring, particularly in future studies with mechanism-specific injury outcomes.

### 4.3. Indicator Combination Effects and Practical Implications

The EWMA four-indicator combination had the highest observed internal AUC (0.958), while the all-12-indicator model had a lower AUC (0.913). This pattern suggests, but does not prove, that adding weakly discriminating or redundant indicators can reduce model performance. Possible mechanisms include noise dilution and feature redundancy across strongly correlated base series. Because feature-importance analyses (for example, permutation importance or SHAP) and formal model comparisons were not available, this explanation is explicitly speculative. With approximately 25 injury events and up to 12 input features, the events-per-variable ratio is about 2:1, so combination-model AUCs should be read as upper-bound internal-validation estimates.

Based on the EWMA four-indicator model, the study proposes a purely conceptual two-tier framework for future research: candidate lower and upper alert boundaries at predicted probabilities > 0.5 and >0.7, respectively. These values were chosen a priori and were not derived using the Youden index, cost-sensitive analysis, calibration assessment, or prospective validation. Under the observed 10.4% injury prevalence, the conventional 0.5 threshold may have poor precision and may not minimize clinically relevant costs. The two-tier framework is therefore not an operational warning system and should not trigger training or medical actions until thresholds are optimized, calibrated, and prospectively validated in independent cohorts.

The sRPE four-indicator combination had a lower observed AUC (0.866) than the EWMA combination but remains a low-cost research candidate because sRPE collection requires only post-session exertion and duration. Its use should still be considered exploratory and contingent on external validation, especially in grassroots programs where analytical capacity and injury-surveillance resources may be limited.

### 4.4. Adolescent Swimming-Specific Considerations

The study population of 13–17-year-old female 200 m individual medley swimmers has developmental characteristics that require cautious interpretation. Skeletal, muscular, cardiorespiratory, and nervous-system maturation may alter workload tolerance and injury susceptibility, but biological maturation was not measured. The earlier statement that an ACWR above 1.5 represents a high-risk zone [31] is based on external literature rather than a threshold derived from this dataset; it should not be interpreted as a validated adolescent swimming cut-off.

Menstrual-cycle phase, hormonal status, menarcheal status, and other maturation markers were not measured. Although existing literature suggests that hormonal fluctuations may influence neuromuscular control, fatigue perception, and recovery, the present data cannot test whether such mechanisms affected RPE variability or injury occurrence [32]. Any hormonal interpretation of the higher observed AUCs for uncoupled ACWR and Diff indicators is therefore speculative and requires direct measurement in future studies.

The 200 m individual medley combines four strokes with high technical complexity and uneven anatomical demands. These event-specific characteristics may limit transportability to sprint or long-distance swimming, where energy-system demands, stroke exposure, and injury patterns differ [33]. The available records did not permit dry-land training to be analyzed as a separate load component, and its contribution to session RPE or injury classification could not be isolated [34]. The present findings should therefore be restricted to female 200 m individual medley swimmers from comparable training contexts until tested in other events and programs.

The findings are directionally consistent with workload-monitoring studies in other youth endurance and power-endurance sports. In champion collegiate cross-country runners, perceived training-dose measures tracked seasonal load meaningfully [28]; in elite youth cricket fast bowlers, weekly load-change metrics performed better than static load levels; and the prevalence of nonfunctional overreaching reported in young English athletes underscores the cost of undetected load surges [16]. This convergence is suggestive rather than evidence that the present thresholds or rankings generalize across sports, events, or injury mechanisms.

Training-phase confounding should also be considered. The 12-week window spanned a general-preparation phase and a pre-competition phase, and the substantial load variability visible in Table 2 (CV approx. 44–56%) partly reflects deliberate phase adjustments rather than random fluctuation [29,30]. Training phase was not included as a covariate, and phase-stratified injury analysis was not performed because per-phase event counts would be too small for stable estimates; whether indicator-injury associations differ across preparation, competition, and taper phases remains an open question for larger datasets.

## 5. Conclusions

In this small single-team sample of female adolescent 200 m individual medley swimmers, sRPE showed moderate-to-substantial standardized agreement with traditional workload indicators, with the distance correlation providing the principal independent evidence and the duration correlation qualified by its part-whole construction. For same-week injury classification, the EWMA four-indicator combination had the highest observed internal AUC (0.958), but this result is preliminary: only 25 injury events were available, athlete-weeks were treated as independent, the classes were imbalanced, injury labels were binary and severity-heterogeneous, AUC differences were not formally tested, confidence intervals overlapped, and no lagged or external validation was performed. EWMA should therefore not yet be preferred over other workload metrics in routine practice. Prospective multi-center studies should benchmark the MLP against penalized logistic regression, test lagged models, account for athlete-level clustering and maturation, evaluate calibration and threshold-dependent metrics, and examine whether adding physiological, biomechanical, or wellness indicators improves performance beyond workload measures alone.

## 6. Practical Applications

The following points derive from a single-team exploratory analysis without lagged or external validation. They are research hypotheses for future investigation, not practice recommendations, clinical decision rules, or validated operational guidance.

sRPE may be evaluated as a low-cost daily workload-monitoring tool in female adolescent 200 m individual medley swimmers, with the distance association providing the strongest independent support. Future studies should test whether sRPE can complement rather than replace objective volume measures because standardized agreement does not imply interchangeability.

EWMA-derived indicators may be investigated further for same-week injury-risk classification. The EWMA four-indicator combination yielded the highest observed internal AUC (0.958), but this value requires lagged, athlete-aware, and external validation before any operational use.

EWMA uncoupled ACWR was the highest-ranked single indicator in this dataset (AUC = 0.946). The numerical patterns favoring uncoupled ACWR and Diff indicators are exploratory because confidence intervals overlapped and formal AUC comparisons were not performed.

A two-tier alert framework could be evaluated in future prospective studies:

A predicted probability threshold of 0.5 could be tested as a candidate lower alert boundary.

A predicted probability threshold of 0.7 could be tested as a candidate upper alert boundary.

These thresholds are conceptual examples only; no coaching action, load modification, or medical referral should be triggered by them until they are optimized, calibrated, and prospectively validated in independent cohorts.

For resource-limited grassroots programs, the sRPE four-indicator combination (AUC = 0.866) is a low-cost candidate for future validation because it requires only post-session RPE and duration recording; it is not yet a validated decision-support tool.

Candidate directions for future research:

Future studies should test whether avoiding rapid week-to-week load increases reduces injury occurrence, while recognizing that the present study establishes association rather than a causal injury trigger.

Future studies should evaluate EWMA as a candidate method for weighting recent loads and reducing day-to-day noise, with confirmation in larger athlete-aware validation studies.

A combined approach that monitors short-term load change (via uncoupled ACWR and Diff) and longer-term load balance (via coupled ACWR) could be tested as a strategy for supporting safe progressive adaptation in youth swimmers.

These research hypotheses may inform future studies of injury-conscious, developmentally appropriate training design. They should not be implemented as practice recommendations until they are prospectively validated across events, sexes, age groups, teams, and sports.

## 7. Research Limitations

1. The sample was small and single-site: 20 female athletes from one provincial youth swimming team, with 240 athlete-weeks and only 25 injury-event weeks (10.4%). This severe imbalance and low event count increase model instability and the likelihood of optimistic internal performance estimates. Bootstrap confidence intervals for several indicators were wide and overlapped.
2. The study was conducted at a single center with one event (200 m individual medley) and one sex. The findings constitute a proof of concept; generalization to male swimmers, sprint or distance events, other age groups, other teams or countries, and non-elite swimmers requires multi-center validation and model transportability testing.
3. Athlete-weeks from the same athlete were treated as independent even though they are nested within athletes. Athlete-specific clustering (training history, injury susceptibility, technique, and maturation) was not modeled, so effective sample size is smaller than the nominal 240, standard errors may be underestimated, and AUC estimates may be optimistic. This limitation is central to interpreting all reported AUCs and confidence intervals. Mixed-effects models, generalized estimating equations, cluster-robust bootstrap resampling, repeated athlete-aware cross-validation, or leave-one-athlete-out validation are needed in future analyses.
4. With 25 injury events and up to 12 input features, the events-per-variable ratio is about 2:1, well below conventional minimums for stable prediction modeling and especially restrictive for neural networks. Neural-network AUCs should therefore be read as upper-bound internal-validation estimates. Penalized logistic regression and other simpler benchmarks were not fitted, so added value over parsimonious models remains unknown; direct comparison with penalized logistic regression is a priority for future reanalysis.
5. All model validation was internal (one 70/30 hold-out plus Bootstrap). No external or independent validation cohort was available, and repeated k-fold cross-validation, cluster-robust resampling, or leave-one-athlete-out validation was not performed; the models must be regarded as preliminary until prospectively validated in independent samples.
6. A major limitation is that injury outcomes were modeled within the same week as the load indicators. The analysis is therefore concurrent classification, not true prospective prediction of future injury. A lagged analysis (week N indicators predicting week N+1 injuries) is required to establish clinical early-warning utility and to reduce concerns about reverse causality.
7. Model evaluation relied on AUC, which summarizes rank-based discrimination but is insufficient for imbalanced classification. Calibration plots, Brier score, precision-recall curves, precision, recall, F1 score, and confusion-matrix metrics were not assessed; no resampling or class weighting was applied. The alert thresholds were not optimized using the Youden index or cost-sensitive analysis. Formal feature-importance analysis and statistical comparison of AUC values (for example, DeLong tests) were also not performed.
8. Injury recording did not include severity grading, time-loss duration, affected tissue, or acute-versus-overuse mechanism, and per-athlete injury distributions were not reported because small cell sizes could risk identifiability within a single named team. The combined medical-attention/time-loss definition creates a heterogeneous outcome, and minor complaints managed without formal medical consultation may not have been captured; the observed 10.4% athlete-week injury rate is therefore a lower-bound estimate.
9. Although a one-week familiarization protocol was completed, formal test-retest reliability of RPE reporting was not quantified. Inter-individual differences in RPE interpretation and reporting maturity may therefore have affected the validity of all downstream workload and classification analyses. A longer familiarization period and post-hoc reliability checks using sessions with similar external loads should be considered.
10. The 12-week duration covered a general-preparation and pre-competition mesocycle but not seasonal variation or longer-term adaptation. Training phase was not modeled and phase-specific sensitivity analyses were not feasible because per-phase event counts were too small.
11. sRPE is a subjective assessment tool and may be affected by adolescent athletes’ perceptual maturity, emotional state, social desirability, and unmeasured biological maturation, despite standardized collection procedures.
12. No direct maturation measures (peak height velocity, menarcheal status, Tanner stage, or longitudinal height change) were collected, and no proxy maturation variable was available for covariate adjustment. Maturation-related variability may therefore confound both workload tolerance and RPE reporting and limit generalizability across the 13–17-year age range.
13. The amount and pattern of missing data were not formally analyzed, and sensitivity analyses using alternative missing-data assumptions were not performed. Assigning zero load or zero REDI weight to missing days avoids imputation but may attenuate cumulative-load estimates if missingness is related to injury, recovery, or unrecorded training.
14. Training duration excluded warm-up and cool-down, whereas some published sRPE protocols include these components; this difference may affect comparability and underestimate total exposure. Dry-land training was not separately quantified, so its contribution to session RPE and injury classification could not be isolated.

## Figures and Tables

**Table 2:** Descriptive statistics of primary variables (N = 261).

Variable	Mean	SD	Min	Max	CV (%)
Training distance (m)	8517	4045	2000	18,500	47.5
Training duration (min)	105	46	40	210	43.7
sRPE (a.u.)	908	512	120	2100	56.4
EWMA (a.u.)	878	409	180	1850	46.6
REDI (a.u.)	3240	1520	620	7800	46.9

**Table 3:** Correlation matrix of training volume and intensity indicators. Note: ** *p* < 0.01 (two-tailed). Values in square brackets are Fisher z 95% confidence intervals (N = 261).

	sRPE	EWMA	REDI
Training distance	0.965 ** [0.956, 0.972]	0.938 ** [0.922, 0.951]	0.734 ** [0.673, 0.785]
Training duration	0.972 ** [0.964, 0.978]	0.956 ** [0.944, 0.965]	0.782 ** [0.730, 0.825]

**Table 4:** Twelve single-indicator MLP same-week injury classification results (ranked by AUC).

Rank	Indicator	AUC	SE	95% CI Lower	95% CI Upper	AUC Category
1	EWMA uncoupled ACWR	0.946	0.031	0.886	1.000	High
2	EWMA Diff	0.906	0.040	0.828	0.984	High
3	sRPE uncoupled ACWR	0.895	0.043	0.811	0.979	High
4	EWMA coupled ACWR	0.869	0.047	0.777	0.961	High
5	REDI coupled ACWR	0.843	0.052	0.741	0.945	High
6	sRPE Diff	0.822	0.054	0.716	0.928	High
7	REDI uncoupled ACWR	0.796	0.057	0.684	0.908	Moderate
8	sRPE coupled ACWR	0.645	0.070	0.508	0.782	Moderate
9	REDI Diff	0.613	0.071	0.474	0.752	Moderate
10	EWMA SD	0.587	0.072	0.446	0.728	Weak
11	sRPE SD	0.436	0.073	0.293	0.579	Weak
12	REDI SD	0.418	0.072	0.277	0.559	Weak

## Data Availability

The original contributions presented in the study are included in the article. Further inquiries can be directed to the corresponding author.
